# Correction: Synthesis and structural characterization of CO_2_-soluble oxidizers [Bu_4_N]BrO_3_ and [Bu_4_N]ClO_3_ and their dissolution in cosolvent-modified CO_2_ for reservoir applications

**DOI:** 10.1039/d1ra90002a

**Published:** 2021-01-25

**Authors:** Katherine L. Hull, Desmond E. Schipper, Allen G. Oliver

**Affiliations:** Aramco Services Company: Aramco Research Center – Houston 16300 Park Row Houston TX 77084 USA katherine.hull@aramcoamericas.com; The Department of Chemistry and Biochemistry, University of Notre Dame Notre Dame Indiana 46556 USA

## Abstract

Correction for ‘Synthesis and structural characterization of CO_2_-soluble oxidizers [Bu_4_N]BrO_3_ and [Bu_4_N]ClO_3_ and their dissolution in cosolvent-modified CO_2_ for reservoir applications’ by Katherine L. Hull *et al.*, *RSC Adv.*, 2020, **10**, 44973–44980, DOI: 10.1039/D0RA09563J.

The authors regret that the value for the solubility of [Bu_4_N]BrO_3_ in the last sentence of the Results and discussion section was given incorrectly.

In the sentence beginning “Notably, the solubility of [Bu_4_N]BrO_3_ achieved…” on page 44978, the corrected sentence should read “Notably, the solubility of [Bu_4_N]BrO_3_ achieved (>0.12 wt%) with ethanol cosolvent significantly exceeds the typical concentrations utilized in the application (∼0.03 wt%)”.

The Royal Society of Chemistry apologises for these errors and any consequent inconvenience to authors and readers.

## Supplementary Material

